# Supplementary material for: A Quinoxaline−Naphthaldehyde Conjugate for Colorimetric Determination of Copper Ion
Source: Molecules. 2022 May 3;27(9):2908. doi: 10.3390/molecules27092908 (PMC9105850; doi:10.3390/molecules27092908)

checkCIF/PLATON report

You have not supplied any structure factors. As a result the full set of tests cannot be run.

THIS REPORT IS FOR GUIDANCE ONLY. IF USED AS PART OF A REVIEW PROCEDURE FOR PUBLICATION, IT SHOULD NOT REPLACE THE EXPERTISE OF AN EXPERIENCED CRYSTALLOGRAPHIC REFEREE.

No syntax errors found. CIF dictionary Interpreting this report

Datablock: QNH

```
Bond precision:   C-C = 0.0096 A          Wavelength=0.71073

Cell:               a=16.455(2)      b=16.455(2)      c=33.088(9)
                    alpha=90         beta=90          gamma=90
Temperature:        296 K

                Calculated                     Reported
Volume          8959(3)                        8959(3)
Space group     I 41/a                         I 41/a
Hall group      -I 4ad                         -I 4ad
Moiety formula  2(C26 H16 N4 O), C H Cl3       ?
Sum formula     C53 H33 Cl3 N8 O2              C26.50 H16.50 Cl1.50 N4 O
Mr              920.22                         460.11
Dx,g cm-3       1.365                          1.365
Z               8                              16
Mu (mm-1)       0.258                          0.258
F000            3792.0                         3792.0
F000'           3796.83
h,k,lmax        18,18,37                       18,18,37
Nref            3434                           3383
Tmin,Tmax       0.952,0.970                    0.952,0.970
Tmin'           0.952

Correction method= # Reported T Limits: Tmin=0.952 Tmax=0.970
AbsCorr = MULTI-SCAN

Data completeness= 0.985           Theta(max)= 23.799

                                                   wR2(reflections)=
R(reflections)= 0.1034( 1630)                      0.3480( 3383)

S = 1.083                    Npar= 319
```

The following ALERTS were generated. Each ALERT has the format
test-name_ALERT_alert-type_alert-level.
Click on the hyperlinks for more details of the test.

Alert level A

```
PLAT355_ALERT_3_A Long   O-H (X0.82,N0.98A)  O1       - H1       .       1.26 Ang.
```

Alert level B

```
THETM01_ALERT_3_B  The value of sine(theta_max)/wavelength is less than 0.575
            Calculated sin(theta_max)/wavelength =    0.5678
PLAT230_ALERT_2_B Hirshfeld Test Diff for    N4       --C20      .        7.2 s.u.
PLAT230_ALERT_2_B Hirshfeld Test Diff for    C10      --C11      .        7.7 s.u.
```

Alert level C

```
PLAT026_ALERT_3_C Ratio Observed / Unique Reflections (too) Low ..        48% Check
PLAT084_ALERT_3_C High wR2 Value (i.e. > 0.25) ...................       0.35 Report
PLAT230_ALERT_2_C Hirshfeld Test Diff for    C4       --C5       .        7.0 s.u.
PLAT230_ALERT_2_C Hirshfeld Test Diff for    C8       --C9       .        7.0 s.u.
PLAT230_ALERT_2_C Hirshfeld Test Diff for    C9       --C10      .        7.0 s.u.
PLAT230_ALERT_2_C Hirshfeld Test Diff for    C13      --C14      .        5.5 s.u.
PLAT230_ALERT_2_C Hirshfeld Test Diff for    C19      --C26      .        6.3 s.u.
PLAT230_ALERT_2_C Hirshfeld Test Diff for    C21      --C22      .        6.2 s.u.
PLAT234_ALERT_4_C Large Hirshfeld Difference C1       --C2       .       0.16 Ang.
PLAT234_ALERT_4_C Large Hirshfeld Difference C15      --C16      .       0.17 Ang.
PLAT241_ALERT_2_C High   'MainMol' Ueq as Compared to Neighbors of        C22 Check
PLAT242_ALERT_2_C Low    'MainMol' Ueq as Compared to Neighbors of        C10 Check
PLAT260_ALERT_2_C Large Average Ueq of Residue Including      Cl1      0.176 Check
PLAT336_ALERT_2_C Long Bond Distance for ..... C30      -Cl3          2.000 Ang.
PLAT340_ALERT_3_C Low Bond Precision on  C-C Bonds ...............    0.00961 Ang.
PLAT481_ALERT_4_C Long D...A H-Bond Reported O1       ..N2       .       3.88 Ang.
```

Alert level G

```
PLAT003_ALERT_2_G Number of Uiso or Uij Restrained non-H Atoms ...          2 Report
PLAT045_ALERT_1_G Calculated and Reported Z Differ by a Factor ...      0.500 Check
PLAT066_ALERT_1_G Predicted and Reported Tmin&Tmax Range Identical          ? Check
PLAT186_ALERT_4_G The CIF-Embedded .res File Contains ISOR Records          1 Report
PLAT300_ALERT_4_G Atom Site Occupancy of Cl1        Constrained at        0.5 Check
PLAT300_ALERT_4_G Atom Site Occupancy of Cl2        Constrained at        0.5 Check
PLAT300_ALERT_4_G Atom Site Occupancy of Cl3        Constrained at        0.5 Check
PLAT300_ALERT_4_G Atom Site Occupancy of C30        Constrained at        0.5 Check
PLAT300_ALERT_4_G Atom Site Occupancy of H30        Constrained at        0.5 Check
PLAT302_ALERT_4_G Anion/Solvent/Minor-Residue Disorder (Resd  2  )       100% Note
PLAT304_ALERT_4_G Non-Integer Number of Atoms in ..... (Resd  2  )       2.50 Check
PLAT432_ALERT_2_G Short Inter X...Y Contact  Cl3      ..C30      .       2.25 Ang.
                                               -x,-1/2-y,z  =      4_545 Check
PLAT480_ALERT_4_G Long H...A H-Bond Reported H1       ..N2       .       2.72 Ang.
PLAT779_ALERT_4_G Suspect or Irrelevant (Bond) Angle(s) in CIF ...      25.00 Deg.
              CL3  -CL3  -C30     4_545   1_555   4_545 ......     #   85 Check
PLAT790_ALERT_4_G Centre of Gravity not Within Unit Cell: Resd.  #          2 Note
              C H Cl3
PLAT860_ALERT_3_G Number of Least-Squares Restraints ............         12 Note
```

```
PLAT967_ALERT_5_G Note: Two-Theta Cutoff Value in Embedded .res ..       50.0 Degree
```

```
  1 ALERT level A = Most likely a serious problem - resolve or explain
  3 ALERT level B = A potentially serious problem, consider carefully
 16 ALERT level C = Check. Ensure it is not caused by an omission or oversight
 17 ALERT level G = General information/check it is not something unexpected

  2 ALERT type 1 CIF construction/syntax error, inconsistent or missing data
 14 ALERT type 2 Indicator that the structure model may be wrong or deficient
  6 ALERT type 3 Indicator that the structure quality may be low
 14 ALERT type 4 Improvement, methodology, query or suggestion
  1 ALERT type 5 Informative message, check
```

It is advisable to attempt to resolve as many as possible of the alerts in all categories. Often the minor alerts point to easily fixed oversights, errors and omissions in your CIF or refinement strategy, so attention to these fine details can be worthwhile. In order to resolve some of the more serious problems it may be necessary to carry out additional measurements or structure refinements. However, the purpose of your study may justify the reported deviations and the more serious of these should normally be commented upon in the discussion or experimental section of a paper or in the "special_details" fields of the CIF. checkCIF was carefully designed to identify outliers and unusual parameters, but every test has its limitations and alerts that are not important in a particular case may appear. Conversely, the absence of alerts does not guarantee there are no aspects of the results needing attention. It is up to the individual to critically assess their own results and, if necessary, seek expert advice.

Publication of your CIF in IUCr journals

A basic structural check has been run on your CIF. These basic checks will be run on all CIFs submitted for publication in IUCr journals (*Acta Crystallographica*, *Journal of Applied Crystallography*, *Journal of Synchrotron Radiation*); however, if you intend to submit to *Acta Crystallographica Section C* or *E* or *IUCrData*, you should make sure that full publication checks are run on the final version of your CIF prior to submission.

Publication of your CIF in other journals

Please refer to the *Notes for Authors* of the relevant journal for any special instructions relating to CIF submission.

PLATON version of 19/02/2022; check.def file version of 19/02/2022

Datablock QNH - ellipsoid plot

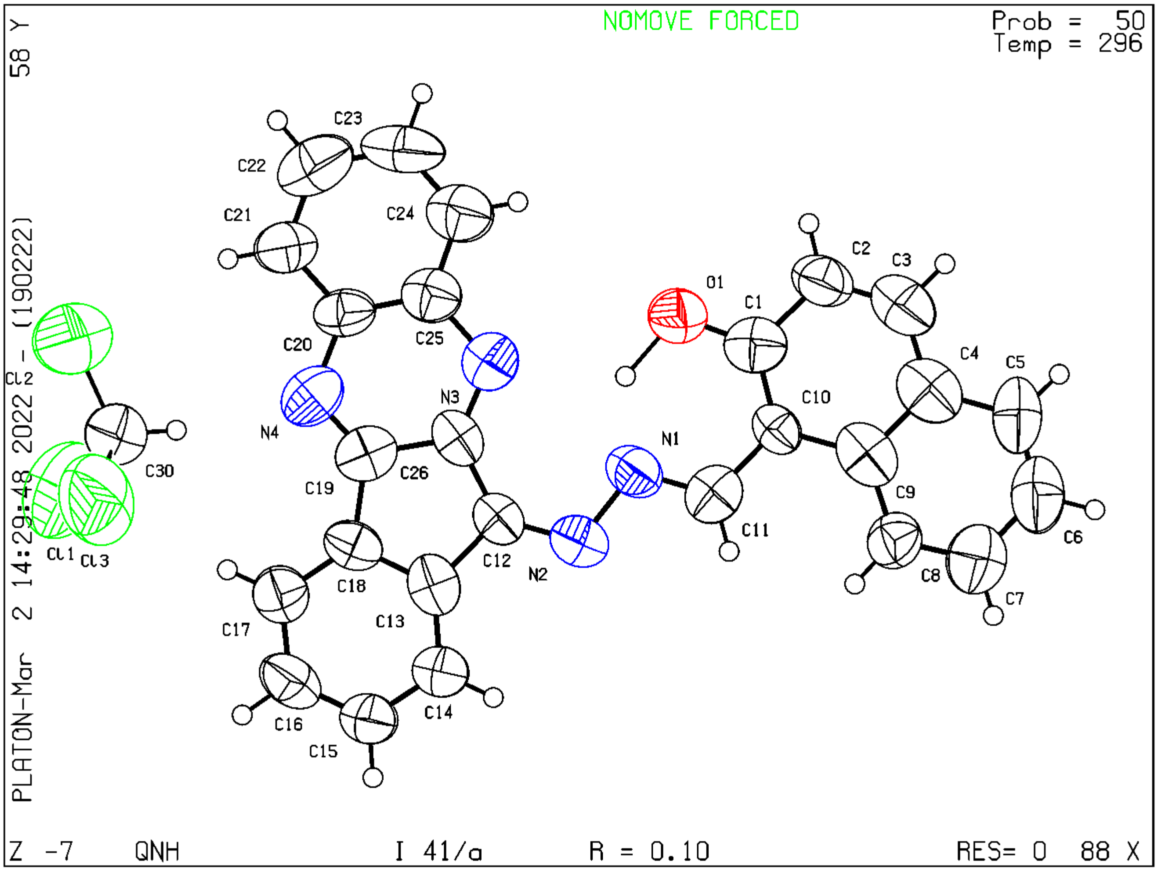

Supplement: Supplementary file 1 [file molecules-27-02908-s001.zip › checkcif_2156635.pdf]
