# Supplementary material for: A Quinoxaline−Naphthaldehyde Conjugate for Colorimetric Determination of Copper Ion
Source: Molecules. 2022 May 3;27(9):2908. doi: 10.3390/molecules27092908 (PMC9105850; doi:10.3390/molecules27092908)

checkCIF/PLATON report

You have not supplied any structure factors. As a result the full set of tests cannot be run.

THIS REPORT IS FOR GUIDANCE ONLY. IF USED AS PART OF A REVIEW PROCEDURE FOR PUBLICATION, IT SHOULD NOT REPLACE THE EXPERTISE OF AN EXPERIENCED CRYSTALLOGRAPHIC REFEREE.

No syntax errors found.   CIF dictionary   Interpreting this report

Datablock: QNHCu

Bond precision: C-C = 0.0231 A   Wavelength=0.71073

Cell:	a=14.067(4)	b=15.587(4)	c=15.601(4)
	alpha=106.091(7)	beta=91.734(7)	gamma=100.266(8)
Temperature:	296 K		

	Calculated	Reported
Volume	3222.6(15)	3222.4(14)
Space group	P -1	P -1
Hall group	-P 1	-P 1
Moiety formula	4(C32 H29 Cu N6 O3), 3(Cl O4), 2(O)	?
Sum formula	C128 H116 Cl3 Cu4 N24 O26	C32 H30 Cl0.75 Cu N6 O6.50
Mr	2767.00	692.75
Dx,g cm-3	1.426	1.428
Z	1	4
Mu (mm-1)	0.795	0.795
F000	1427.0	1431.0
F000'	1429.27	
h,k,lmax	16,18,18	16,18,18
Nref	11366	11126
Tmin,Tmax	0.853,0.916	0.853,0.916
Tmin'	0.853	

Correction method= # Reported T Limits: Tmin=0.853 Tmax=0.916
AbsCorr = MULTI-SCAN

Data completeness= 0.979   Theta(max)= 24.999

R(reflections)= 0.1842(7896)   wR2(reflections)= 0.5019(11126)

S = 2.051   Npar= 838

The following ALERTS were generated. Each ALERT has the format
test-name_ALERT_alert-type_alert-level.
Click on the hyperlinks for more details of the test.

Alert level A

```
PLAT084_ALERT_3_A High wR2 Value (i.e. > 0.25) ...................       0.50 Report
```

Alert level B

```
PLAT082_ALERT_2_B High R1 Value ..................................       0.18 Report
PLAT097_ALERT_2_B Large Reported Max.  (Positive) Residual Density       3.32 eA-3
PLAT306_ALERT_2_B Isolated Oxygen Atom (H-atoms Missing ?) .......        O1W Check
PLAT341_ALERT_3_B Low Bond Precision on  C-C Bonds ...............    0.02312 Ang.
```

Alert level C

```
DIFMX02_ALERT_1_C  The maximum difference density is > 0.1*ZMAX*0.75
            The relevant atom site should be identified.
GOODF01_ALERT_2_C  The least squares goodness of fit parameter lies
            outside the range 0.80 <> 2.00
            Goodness of fit given =      2.051
PLAT018_ALERT_1_C _diffrn_measured_fraction_theta_max .NE. *_full           ! Check
PLAT041_ALERT_1_C Calc. and Reported SumFormula    Strings  Differ     Please Check
PLAT043_ALERT_1_C Calculated and Reported Mol. Weight Differ by ..       4.00 Check
PLAT068_ALERT_1_C Reported F000 Differs from Calcd (or Missing)...     Please Check
PLAT087_ALERT_2_C Unsatisfactory S value (Too High) .............       2.05 Check
PLAT094_ALERT_2_C Ratio of Maximum / Minimum Residual Density ....       3.22 Report
PLAT214_ALERT_2_C Atom O1        (Anion/Solvent) ADP max/min Ratio        4.2 prolat
PLAT220_ALERT_2_C NonSolvent   Resd 1   C   Ueq(max)/Ueq(min) Range       4.1 Ratio
PLAT220_ALERT_2_C NonSolvent   Resd 2   C   Ueq(max)/Ueq(min) Range       4.1 Ratio
PLAT222_ALERT_3_C NonSolvent Resd 1   H   Uiso(max)/Uiso(min) Range       4.7 Ratio
PLAT222_ALERT_3_C NonSolvent Resd 2   H   Uiso(max)/Uiso(min) Range       4.7 Ratio
PLAT234_ALERT_4_C Large Hirshfeld Difference N6A      --C30A     .       0.18 Ang.
PLAT234_ALERT_4_C Large Hirshfeld Difference N6B      --C30B     .       0.19 Ang.
PLAT241_ALERT_2_C High   'MainMol' Ueq as Compared to Neighbors of       C16A Check
PLAT242_ALERT_2_C Low    'MainMol' Ueq as Compared to Neighbors of        N5A Check
PLAT242_ALERT_2_C Low    'MainMol' Ueq as Compared to Neighbors of        N6A Check
PLAT242_ALERT_2_C Low    'MainMol' Ueq as Compared to Neighbors of        N5B Check
PLAT242_ALERT_2_C Low    'MainMol' Ueq as Compared to Neighbors of        N6B Check
PLAT260_ALERT_2_C Large Average Ueq of Residue Including       Cl2      0.159 Check
PLAT260_ALERT_2_C Large Average Ueq of Residue Including       Cl1      0.212 Check
PLAT410_ALERT_2_C Short Intra H...H Contact  H8A      ..H11A     .       1.96 Ang.
                                                      x,y,z  =      1_555 Check
PLAT410_ALERT_2_C Short Intra H...H Contact  H8B      ..H11B     .       1.98 Ang.
                                                      x,y,z  =      1_555 Check
PLAT413_ALERT_2_C Short Inter XH3 .. XHn     H7A      ..H32A     .       2.13 Ang.
                                                    x,1+y,z  =      1_565 Check
PLAT601_ALERT_2_C Unit Cell Contains Solvent Accessible VOIDS of .         86 Ang**3
```

Alert level G

```
FORMU01_ALERT_2_G  There is a discrepancy between the atom counts in the
            _chemical_formula_sum and the formula from the _atom_site* data.
            Atom count from _chemical_formula_sum:C32 H30 Cl0.75 Cu1 N6 O6.5
```

```
            Atom count from the _atom_site data:  C32 H29 Cl0.75 Cu1 N6 O6.5
CELLZ01_ALERT_1_G Difference between formula and atom_site contents detected.
CELLZ01_ALERT_1_G WARNING: H atoms missing from atom site list. Is this intentional?
           From the CIF: _cell_formula_units_Z    4
           From the CIF: _chemical_formula_sum  C32 H30 Cl0.75 Cu N6 O6.50
           TEST: Compare cell contents of formula and atom_site data

           atom    Z*formula  cif sites diff
           C        128.00    128.00    0.00
           H        120.00    116.00    4.00
           Cl         3.00      3.00    0.00
           Cu         4.00      4.00    0.00
           N         24.00     24.00    0.00
           O         26.00     26.00    0.00
PLAT003_ALERT_2_G Number of Uiso or Uij Restrained non-H Atoms ...          2 Report
PLAT045_ALERT_1_G Calculated and Reported Z Differ by a Factor ...      0.250 Check
PLAT066_ALERT_1_G Predicted and Reported Tmin&Tmax Range Identical          ? Check
PLAT186_ALERT_4_G The CIF-Embedded .res File Contains ISOR Records          1 Report
PLAT244_ALERT_4_G Low    'Solvent' Ueq as Compared to Neighbors of        Cl1 Check
PLAT300_ALERT_4_G Atom Site Occupancy of O5         Constrained at        0.5 Check
PLAT300_ALERT_4_G Atom Site Occupancy of O6         Constrained at        0.5 Check
PLAT300_ALERT_4_G Atom Site Occupancy of O7         Constrained at        0.5 Check
PLAT300_ALERT_4_G Atom Site Occupancy of O8         Constrained at        0.5 Check
PLAT302_ALERT_4_G Anion/Solvent/Minor-Residue Disorder (Resd  3  )        80% Note
PLAT333_ALERT_2_G Large Aver C6-Ring C-C Dist C1B      -C10B     .       1.42 Ang.
PLAT380_ALERT_4_G Incorrectly? Oriented X(sp2)-Methyl Moiety .....       C28A Check
PLAT380_ALERT_4_G Incorrectly? Oriented X(sp2)-Methyl Moiety .....       C29A Check
PLAT380_ALERT_4_G Incorrectly? Oriented X(sp2)-Methyl Moiety .....       C28B Check
PLAT380_ALERT_4_G Incorrectly? Oriented X(sp2)-Methyl Moiety .....       C29B Check
PLAT721_ALERT_1_G Bond    Calc     0.97000, Rep     0.96000 Dev...       0.01 Ang.
              C32B     -H32B            1_555   1_555 ........     #  155 Check
PLAT779_ALERT_4_G Suspect or Irrelevant (Bond) Angle(s) in CIF ...      42.00 Deg.
              O6   -O8   -CL2     2_766   1_555   1_555 ......     #  313 Check
PLAT794_ALERT_5_G Tentative Bond Valency for Cu1A      (II)      .       2.22 Info
PLAT794_ALERT_5_G Tentative Bond Valency for Cu1B      (II)      .       2.13 Info
PLAT860_ALERT_3_G Number of Least-Squares Restraints ............          12 Note
PLAT870_ALERT_4_G ALERTS Related to Twinning Effects Suppressed ..          ! Info
PLAT941_ALERT_3_G Average HKL Measurement Multiplicity ..........         2.4 Low
PLAT967_ALERT_5_G Note: Two-Theta Cutoff Value in Embedded .res ..       50.0 Degree
```

---

   1 **ALERT level A** = Most likely a serious problem - resolve or explain
   4 **ALERT level B** = A potentially serious problem, consider carefully
  26 **ALERT level C** = Check. Ensure it is not caused by an omission or oversight
  26 **ALERT level G** = General information/check it is not something unexpected

  10 ALERT type 1 CIF construction/syntax error, inconsistent or missing data
  23 ALERT type 2 Indicator that the structure model may be wrong or deficient
   6 ALERT type 3 Indicator that the structure quality may be low
  15 ALERT type 4 Improvement, methodology, query or suggestion
   3 ALERT type 5 Informative message, check

It is advisable to attempt to resolve as many as possible of the alerts in all categories. Often the minor alerts point to easily fixed oversights, errors and omissions in your CIF or refinement strategy, so attention to these fine details can be worthwhile. In order to resolve some of the more serious problems it may be necessary to carry out additional measurements or structure refinements. However, the purpose of your study may justify the reported deviations and the more serious of these should normally be commented upon in the discussion or experimental section of a paper or in the "special_details" fields of the CIF. checkCIF was carefully designed to identify outliers and unusual parameters, but every test has its limitations and alerts that are not important in a particular case may appear. Conversely, the absence of alerts does not guarantee there are no aspects of the results needing attention. It is up to the individual to critically assess their own results and, if necessary, seek expert advice.

Publication of your CIF in IUCr journals

A basic structural check has been run on your CIF. These basic checks will be run on all CIFs submitted for publication in IUCr journals (*Acta Crystallographica*, *Journal of Applied Crystallography*, *Journal of Synchrotron Radiation*); however, if you intend to submit to *Acta Crystallographica Section C* or *E* or *IUCrData*, you should make sure that full publication checks are run on the final version of your CIF prior to submission.

Publication of your CIF in other journals

Please refer to the *Notes for Authors* of the relevant journal for any special instructions relating to CIF submission.

PLATON version of 19/02/2022; check.def file version of 19/02/2022

Datablock QNHCu - ellipsoid plot

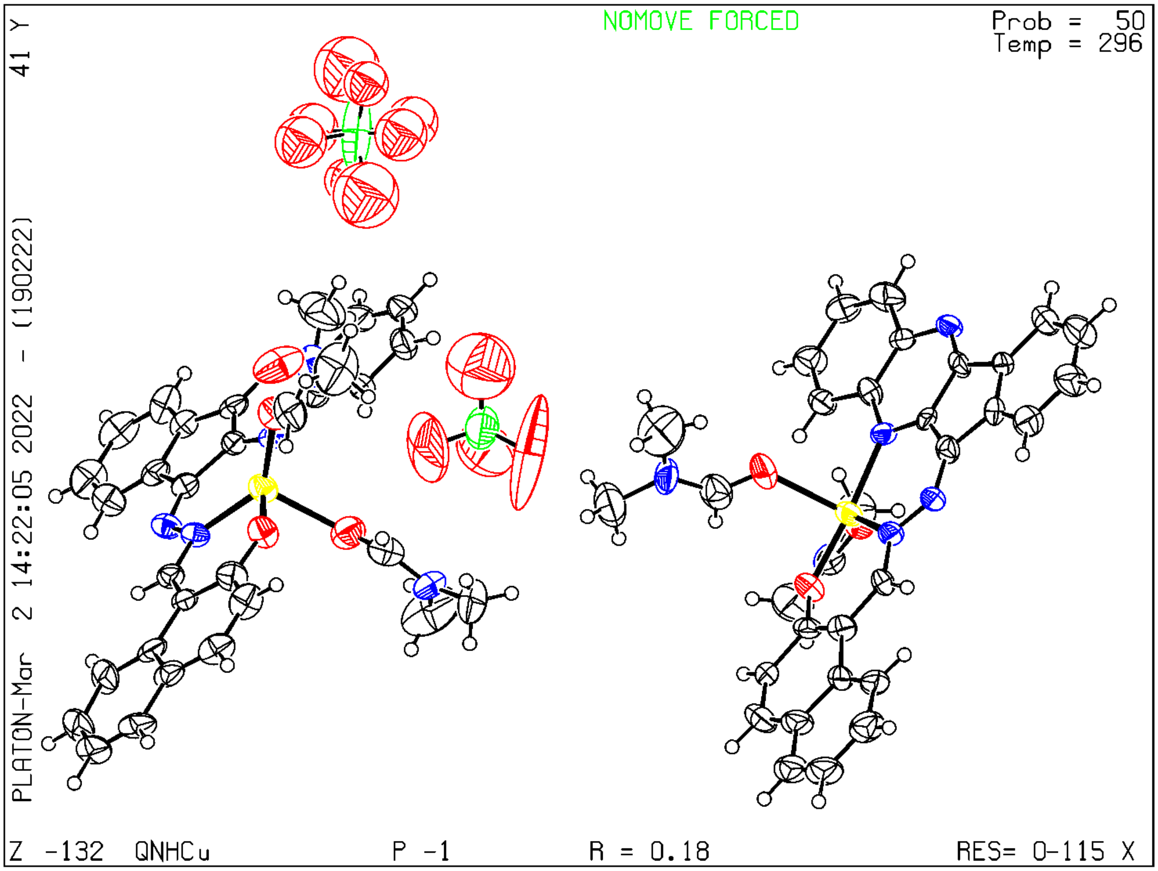

Supplement: Supplementary file 1 [file molecules-27-02908-s001.zip › checkcif_2156636.pdf]
