# Supplementary material for: A Quinoxaline−Naphthaldehyde Conjugate for Colorimetric Determination of Copper Ion
Source: Molecules. 2022 May 3;27(9):2908. doi: 10.3390/molecules27092908 (PMC9105850; doi:10.3390/molecules27092908)
Supplement: Supplementary file 1 [file molecules-27-02908-s001.zip › ESI_molecules-1648806.pdf]

Electronic Supplementary Information

A quinoxaline–naphthaldehyde conjugate for colorimetric determination of copper ion

Sutapa Sahu[1], Yeasin Sikdar[1,2], Riya Bag [1], Michael G. B. Drew [3], José P. Cerón-Carrasco [4,*] and Sanchita Goswami[1,*]

[1] Department of Chemistry, University of Calcutta, 92, A.P.C. Road, Kolkata 700009, India; sutapa.sahu11@gmail.com (S.S.); y.sikdar@gmail.com (Y.S.); riyabag.chem@gmail.com (R.B.)

[2] Department of Chemistry, The Bhawanipur Education Society College, 5, Lala Lajpat Rai Sarani, Kolkata 700020, India; y.sikdar@gmail.com (Y.S.)

[3] Department of Chemistry, University of Reading, Whiteknights, Reading RG6 6AD, UK

[4] Centro Universitario de la Defensa, Academia General del Aire, Universidad Politécnica de Cartagena, C/Coronel López Peña S/N, Santiago de La Ribera, 30720 Murcia, Spain

* Correspondence: jose.ceron@cud.upct.es (J.P.C.-C.); sgchem@caluniv.ac.in (S.G.)

Contents of the Supporting Information

	**Page No.**
**Scheme S1.** Synthetic procedure of QNH.	S3
**Figure S1.** Molecular structure of QNH. Color code: C = pista green; N = blue; O = red; H= gray; Cl = bright green	S4
**Figure S2.** ^{1}H NMR spectrum of QNH in $CDCl_3$.	S4
**Figure S3.** ESI–MS spectrum of QNH.	S5
**Figure S4.** ESI–MS spectrum of QNH + Cu^{2+} complex.	S5
**Figure S5.** FT–IR spectrum of QNH.	S6
**Figure S6.** FT–IR spectrum of QNH + Cu^{2+} complex	S6

**Figure S7.** Absorbance spectra of QNH (10^{-5} M) in presence of 5 equiv. of various cations (Na^{+}, K^{+}, Ca^{2+}, Mg^{2+}, Hg^{2+}, Ni^{2+}, Fe^{3+}, Cu^{2+}, Co^{2+}, Cd^{2+}, Zn^{2+}, Mn^{2+}, Pb^{2+}, Al^{3+}, Cr^{3+}) in (3:7, v/v) HEPES buffer : acetonitrile medium.	S7
**Figure S8.** Absorption intensity of QNH (10^{-5} M) in the presence of 5 equiv. of different cations in absence of same equiv. of Cu^{2+} in solution [the green bar portion]. Absorption intensity of a mixture of QNH (10^{-5} M) with 5 equiv. of other cations followed by addition of 5 equiv. of Cu^{2+} to the solution (λ_{abs}=552 nm) [the orange blue bar portion].	S7
**Figure S9.** Determination of the detection limit of QNH in presence of Cu^{2+} in (3:7 v/v) HEPES buffer : acetonitrile medium at 552 nm.	S8
**Figure S10.** Job′s plot for the identification of stoichiometry using absorbance values at 552 nm.	S8
**Figure S11.** Benesi-Hildebrand plot for determination of binding constant of **QNH** with Cu^{2+}.	S9
**Figure S12.** Absorbance of QNH in absence and in presence of Cu^{2+} at different pH values.	S9
**Figure S13.** Absorbance spectra of QNH in present different anion Cu^{2+} of salts. (Inset: Bar plot of QNH in present different anion Cu^{2+} of salts)	S10
**Figure S14.** (A) Absorption spectrum of QNH shows reversibility towards Cu^{2+} in presence of EDTA in (3:7, v/v) HEPES buffer : acetonitrile medium. (B) The reversible colorimetric switch by alternate addition of $Cu^{2+}$and EDTA into the (3:7, v/v) HEPES buffer : acetonitrile medium. (C) Visual color change of QNH showing reversibility towards Cu^{2+} in presence of EDTA.	S10
**Figure S15.** (A) UV–Visible titration of QNH–Cu^{2+} complex (10^{-5}M) in the presence of various concentration of histidine. (Downward arrow indicates gradual decrease of absorption at 552 nm with increasing concentration of externally added histidine) (Inset: Visual color change of QNH in presence of Cu^{2+} followed by histidine). (B)Absorbance of QNH–Cu^{2+} complex at 552 nm as a function of [histidine]).	S11
**Figure S16.** Determination of the detection limit of Histidine in (3:7 v/v) HEPES buffer :	S11

acetonitrile medium at 552 nm.	
**Figure S17.** Visual change of QNH in presence of Cu^{2+} in A) paper strip and B)gel form.	S12
**Figure S18.** Crystal structure of QNH + Cu^{2+} metal complex. (Inset: The geometry of the two Cu centre of the complex in unit A and unit B). Color code: C = pista green; N = blue; O = red; H= gray; Cl = bright green; Cu = cyan.	S12
**Figure S19.** The supramolecular 2D packing diagram of the QNH showing the propagation via H–bonding and short ring–interactions, forms a 2D sheet like structure.	S13
**Figure S20.** The supramolecular 1D packing diagram of QNH via (A) H–bonding interaction along b axis with enlarged version and c axis view of the interaction; (B) short ring–interactions with enlarged version and c axis view of the interaction.	S13
**Table S1.** Colorimetric Cu^{2+} sensing receptors found in the literature.	S14
**Table S2.** The crystallographic data for QNH.	S16
**Table S3.** The crystallographic data for QNH + Cu^{2+}.	S17
**Table S4.** Analysis of X–I···J (H bonding) interactions in QNH. (Bond lengths (Å), bond angles (°)).	S19
**Table S5.** Analysis of short ring–interactions with Cg···Cg distances (Å) in QNH.	S19

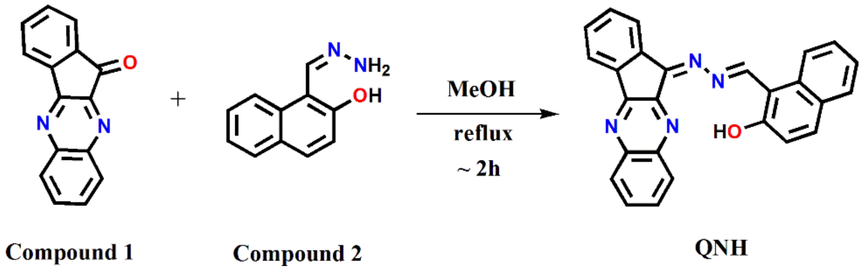

Scheme S1. Synthetic procedure of QNH**.**

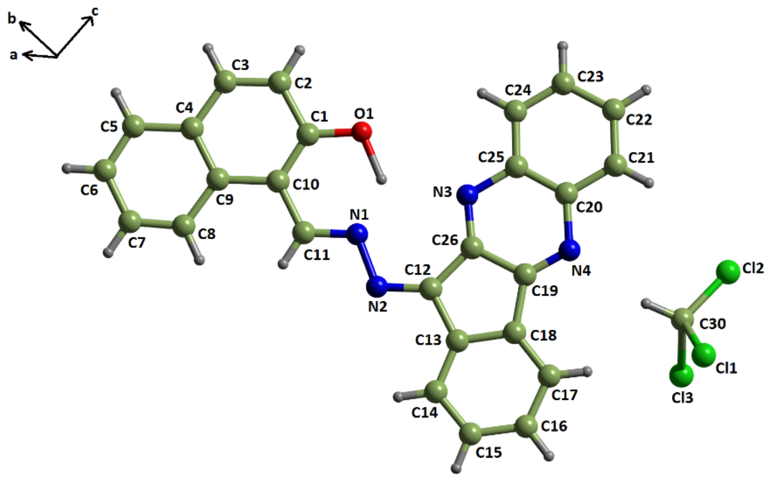

Figure S1. Molecular structure of QNH. Color code: C = pista green; N = blue; O = red; H= gray; Cl = bright green.

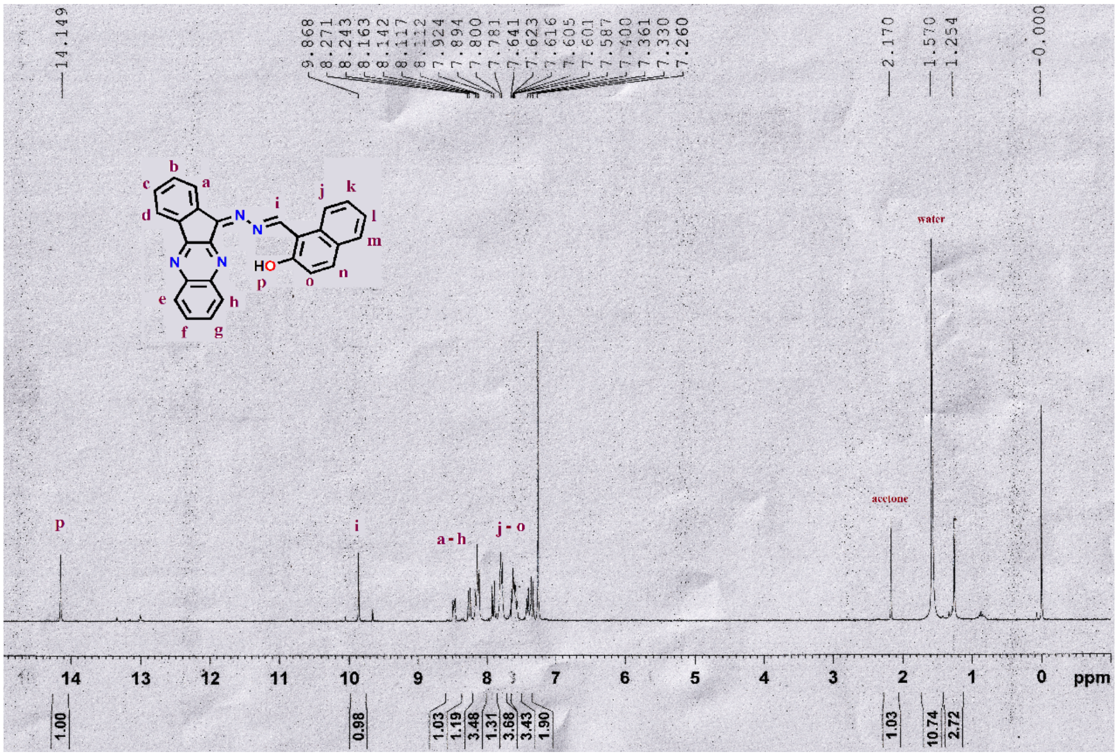

Figure S2. ^{1}H NMR spectrum of QNH in $CDCl_3$.

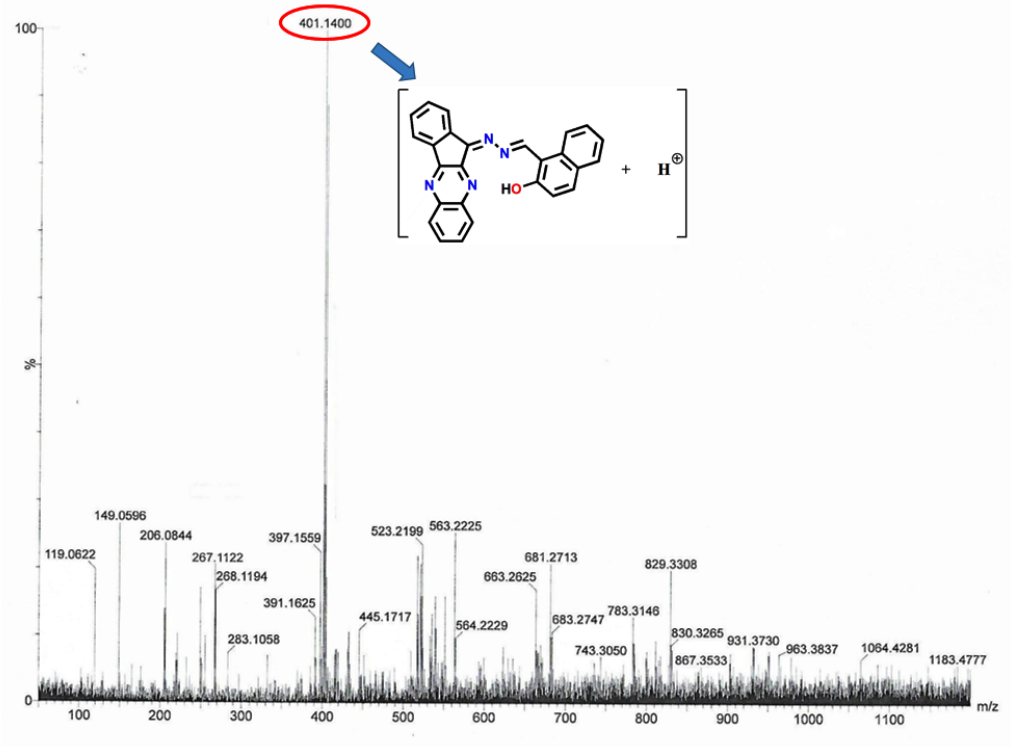

Figure S3. ESI–MS spectrum of QNH.

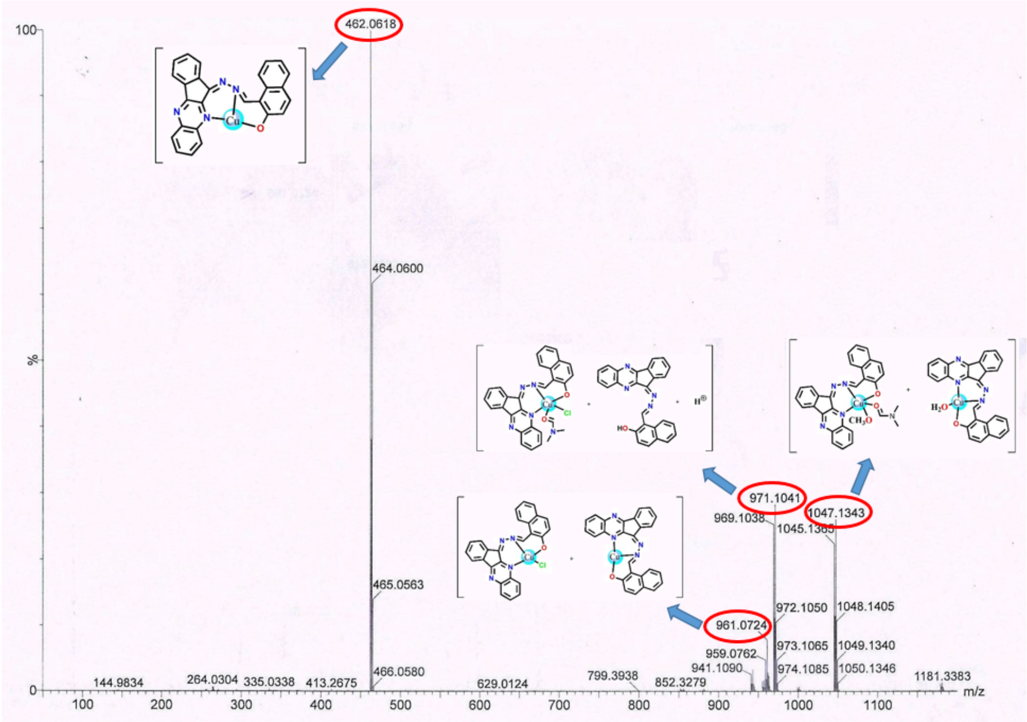

Figure S4. ESI–MS Spectrum of QNH + Cu^{2+} complex.

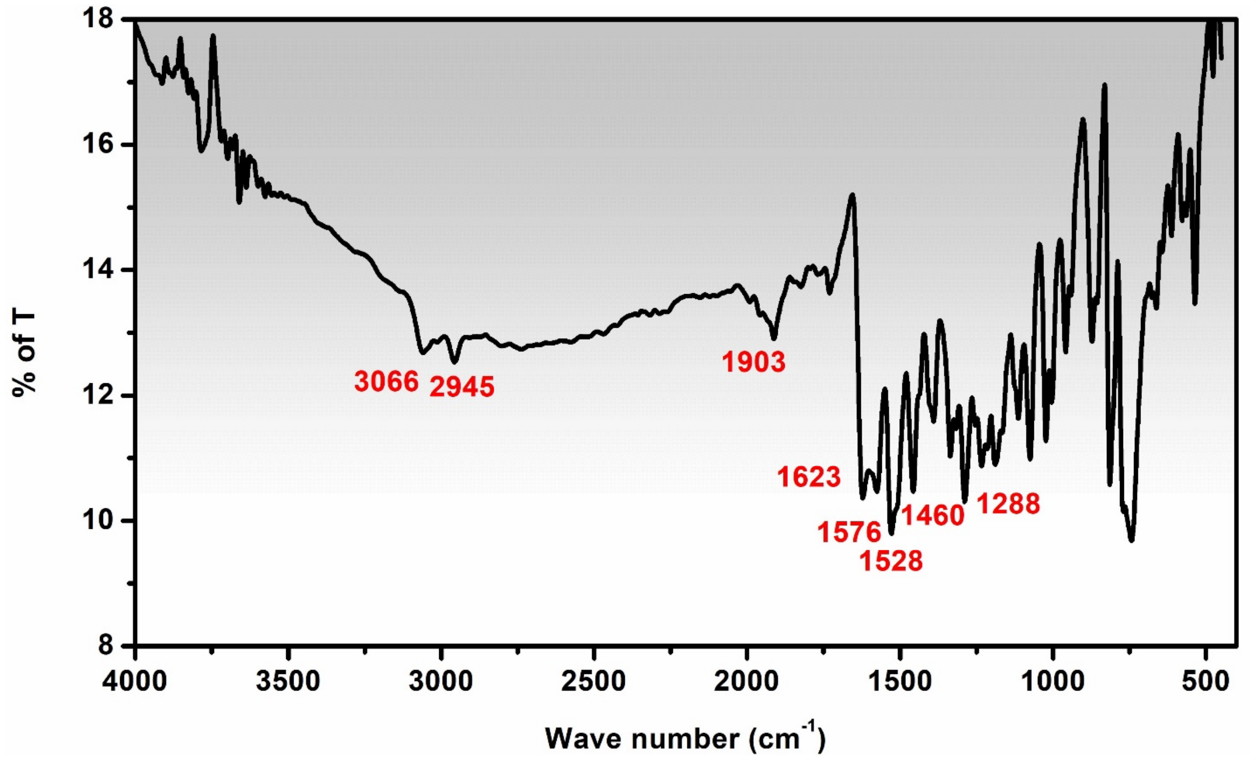

Figure S5. FT–IR Spectrum of QNH.

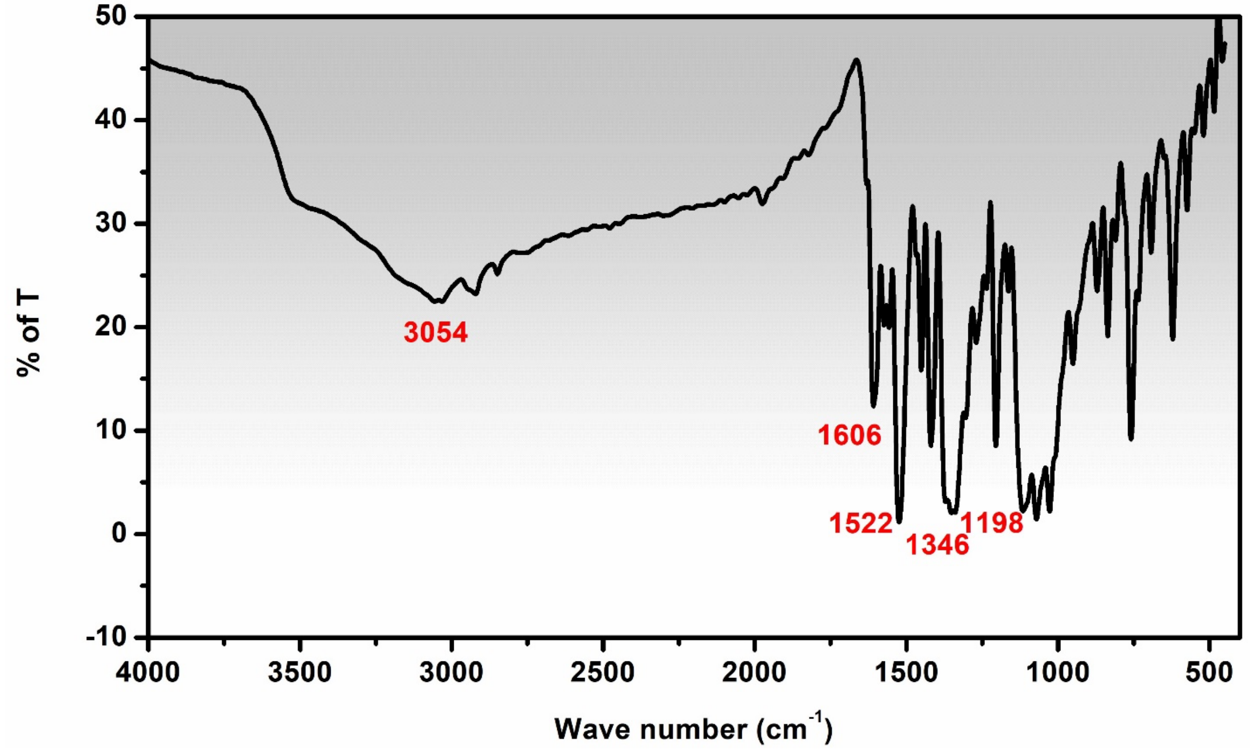

Figure S6. FT–IR Spectrum of QNH + Cu^{2+} complex

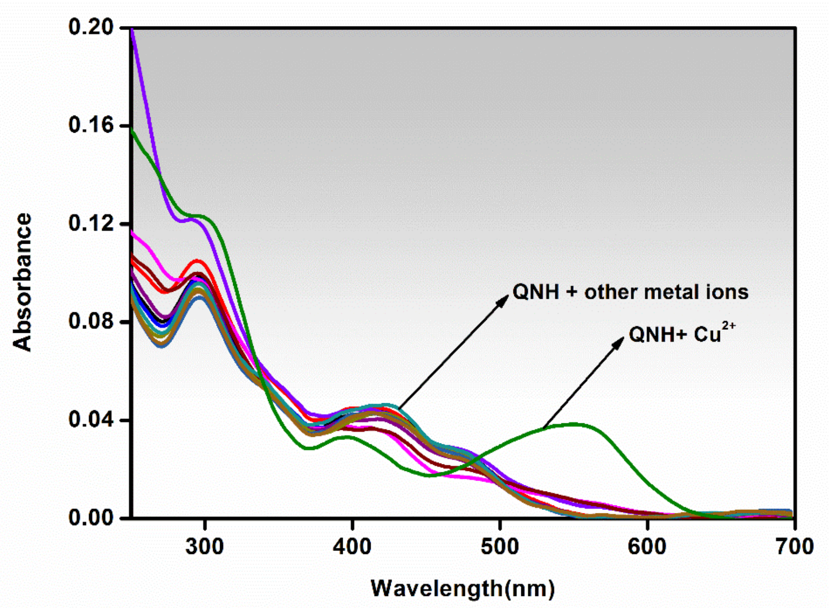

Figure S7. Absorbance spectra of QNH (10^{-5} M) in presence of 5 equiv. of various cations (Na^+, K^+, Ca^{2+}, Mg^{2+}, Hg^{2+}, Ni^{2+}, Fe^{3+}, Cu^{2+}, Co^{2+}, Cd^{2+}, Zn^{2+}, Mn^{2+}, Pb^{2+}, Al^{3+}, Cr^{3+}) in (3:7, v/v)HEPES buffer : acetonitrile medium.

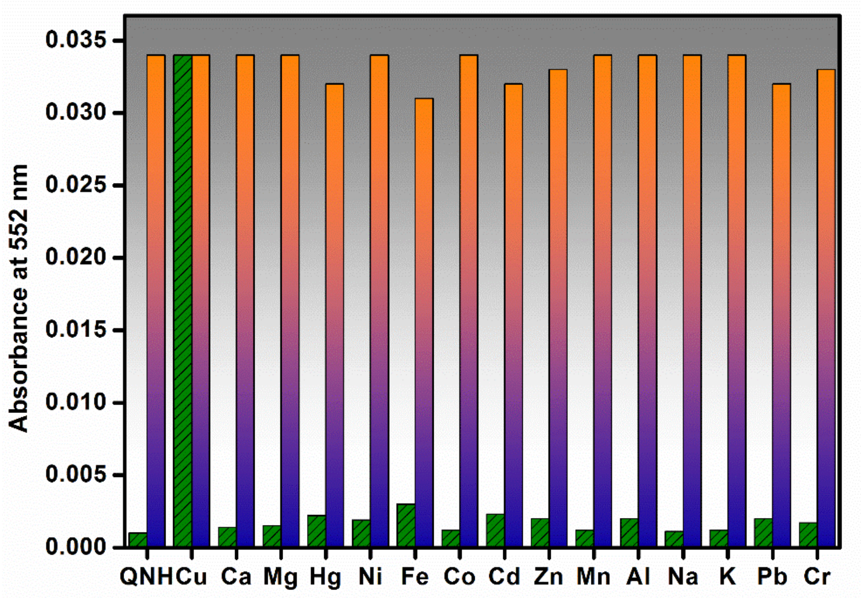

Figure S8. Absorption intensity of QNH (10^{-5} M) in the presence of 5 equiv. of different cations in absence of same equiv. of Cu^{2+} in solution [the green bar portion]. Absorption intensity of a mixture of **QNH** (10^{-5} M) with 5 equiv. of other cations followed by addition of 5 equiv. of Cu^{2+} to the solution (λ_{abs}=552nm) [the orange blue bar portion].

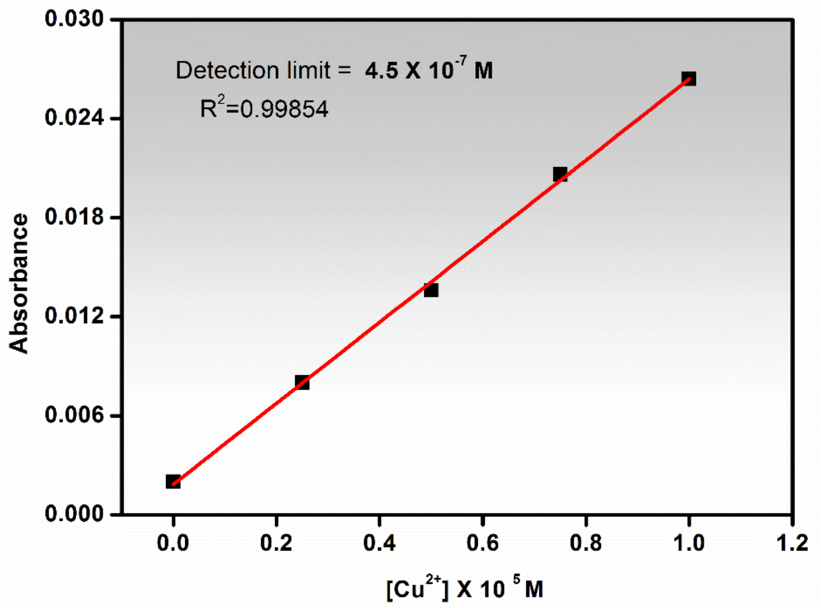

Figure S9. Determination of the detection limit of QNH in presence of Cu^{2+} in (3:7, v/v) HEPES buffer : acetonitrile medium at 552 nm.

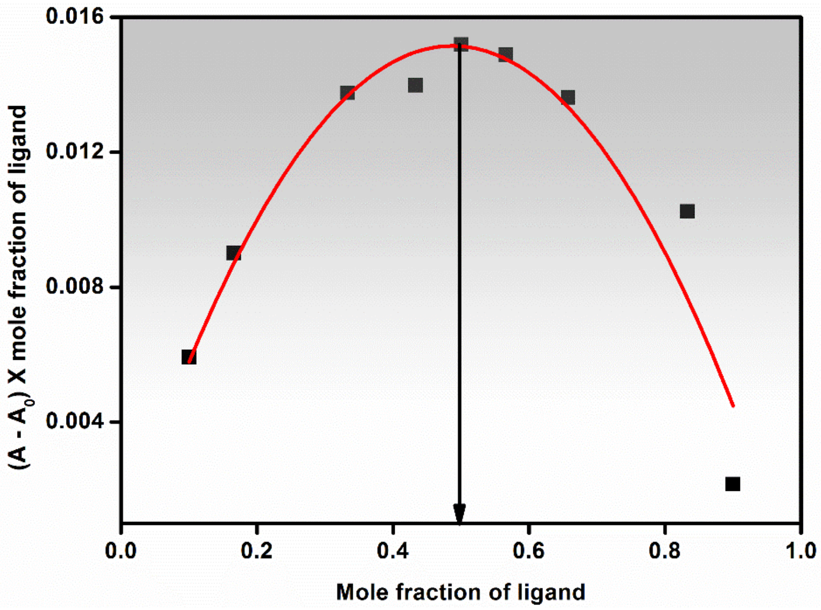

Figure S10. Job's plot for the identification of stoichiometry using absorbance values at 552 nm.

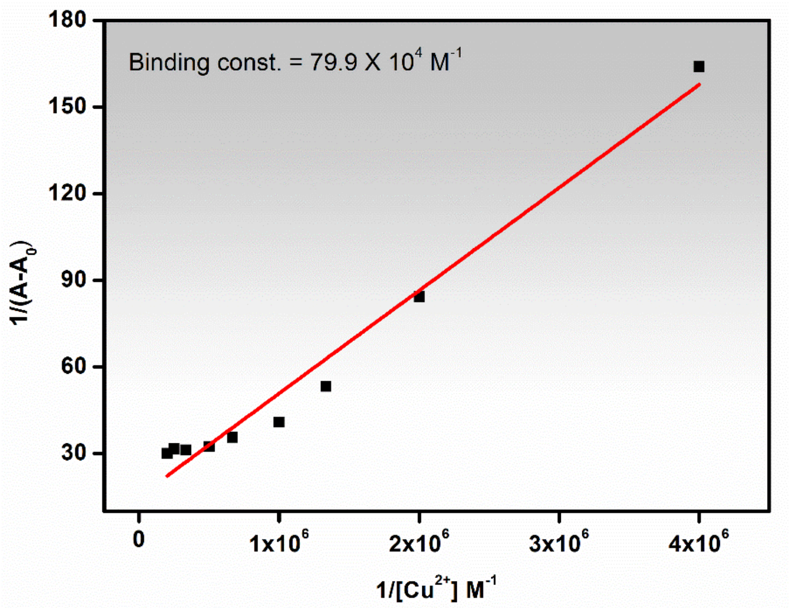

Figure S11. Benesi-Hildebrand plot for determination of binding constant of QNH with Cu^{2+}.

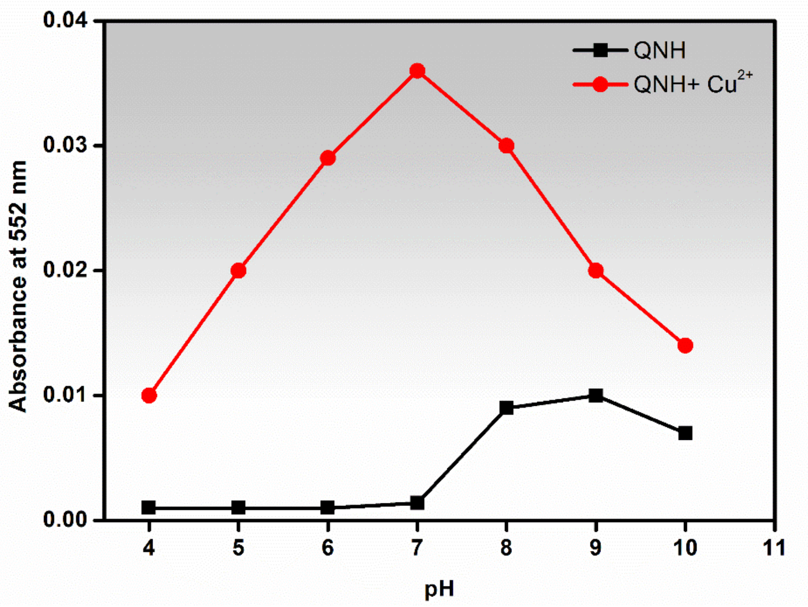

Figure S12. Absorbance of QNH in absence and in presence of Cu^{2+} at different pH values.

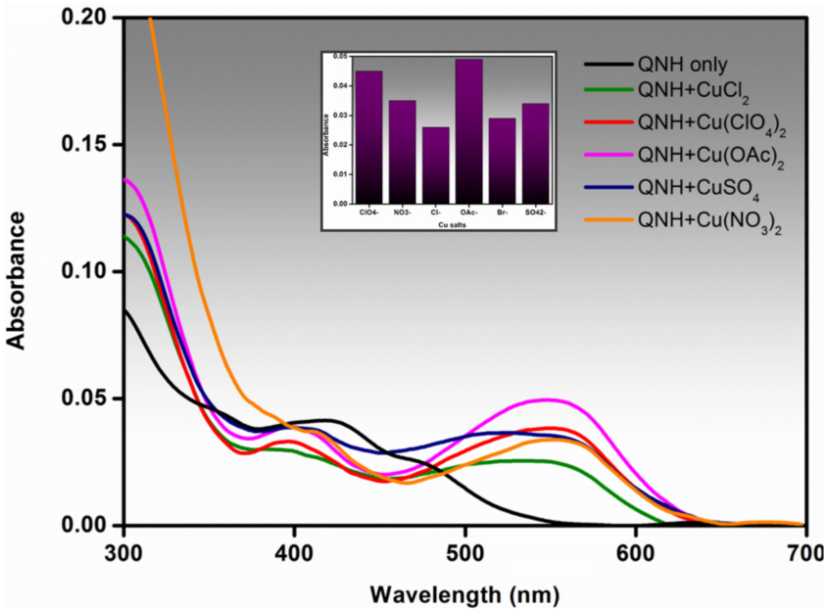

Figure S13. Absorbance spectra of QNH in present different anion Cu^{2+} of salts. (Inset: Bar plot of QNH in present different anion Cu^{2+} of salts)

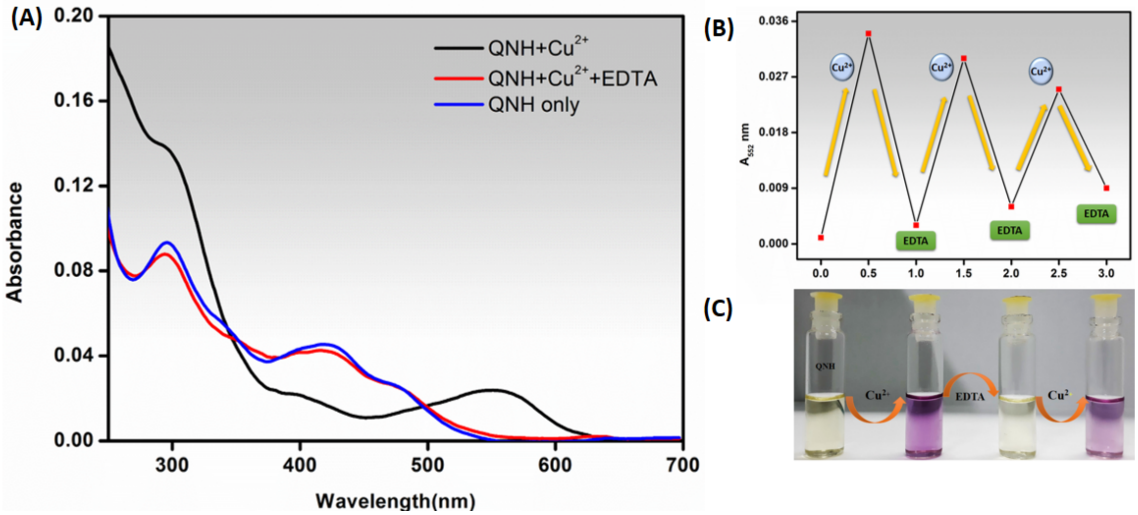

Figure S14. (A) Absorption spectrum of QNH shows reversibility towards Cu^{2+} in presence of EDTA in (3:7, v/v)HEPES buffer : acetonitrile medium. (B) The reversible colorimetric switch by alternate addition of $Cu^{2+}$and EDTA into the (3:7, v/v)HEPES buffer : acetonitrile medium. (C) Visual color change of **QNH** showing reversibility towards Cu^{2+} in presence of EDTA.

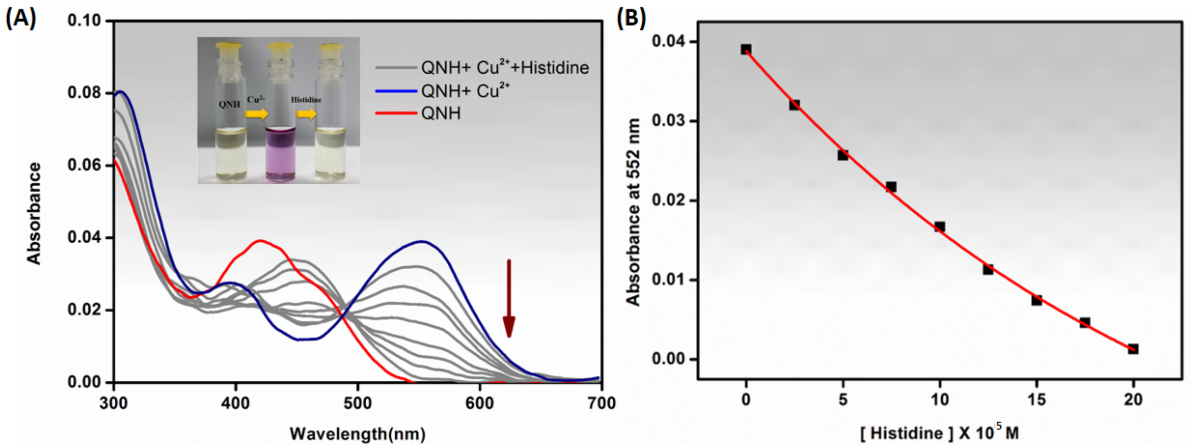

Figure S15. (A)UV–Vis titration of QNH–Cu^{2+} complex (10^{-5}M) in the presence of various concentration of Histidine. (Downward arrow indicates gradual decrease of absorption at 552 nm with increasing concentration of externally added Histidine) (Inset: Visual color change of QNH in presence of Cu^{2+} followed by histidine). (B)Absorbance of QNH–Cu^{2+} complex at 552 nm as a function of [Histidine]).

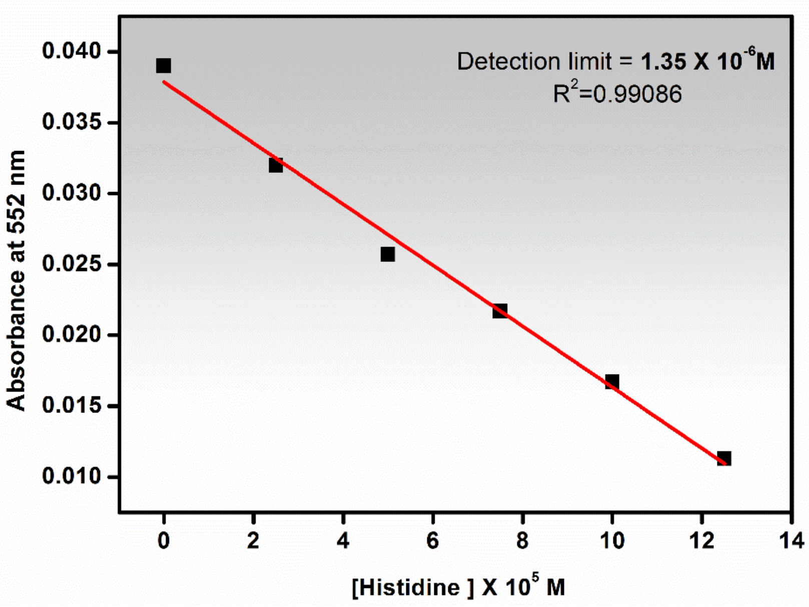

Figure S16. Determination of the detection limit of Histidine in (3:7 v/v)HEPES buffer : acetonitrile medium at 552 nm.

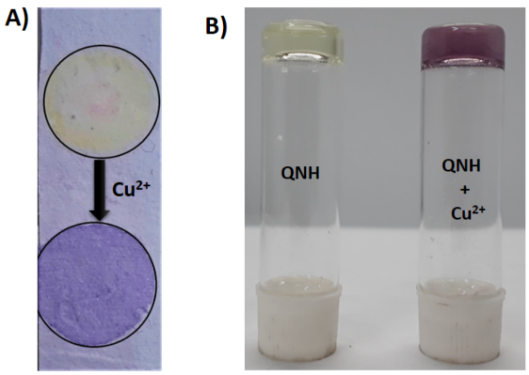

Figure S17. Visual change of QNH in presence of Cu^{2+} in A) paper strip and B)gel form.

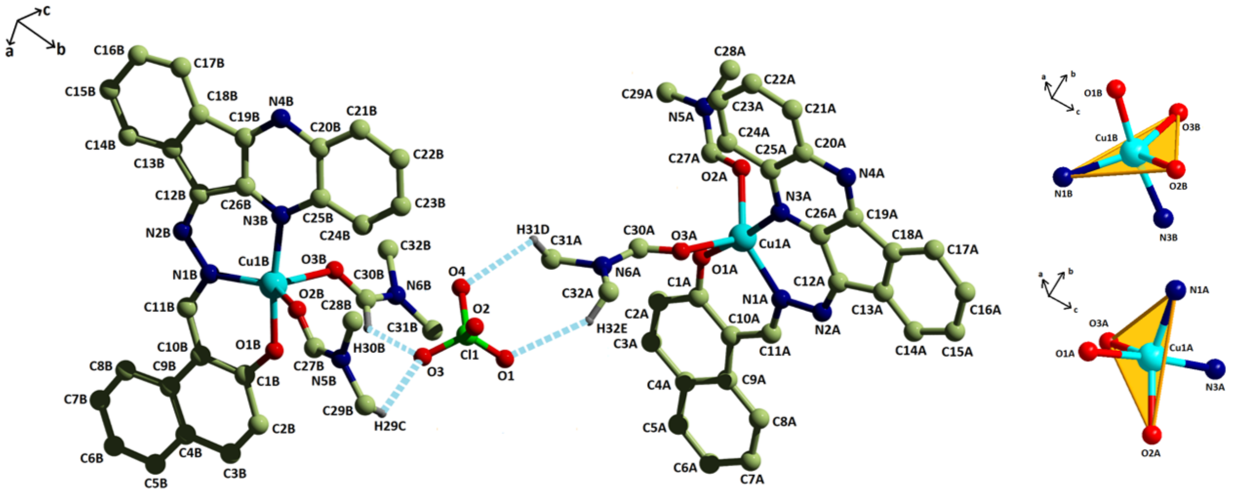

Figure S18. Crystal structure of QNH + Cu^{2+} metal complex. (Inset: The geometry of the two Cu centre of the complex in unit A and unit B). Color code: C = pista green; N = blue; O = red; H= gray; Cl = bright green; Cu = cyan.

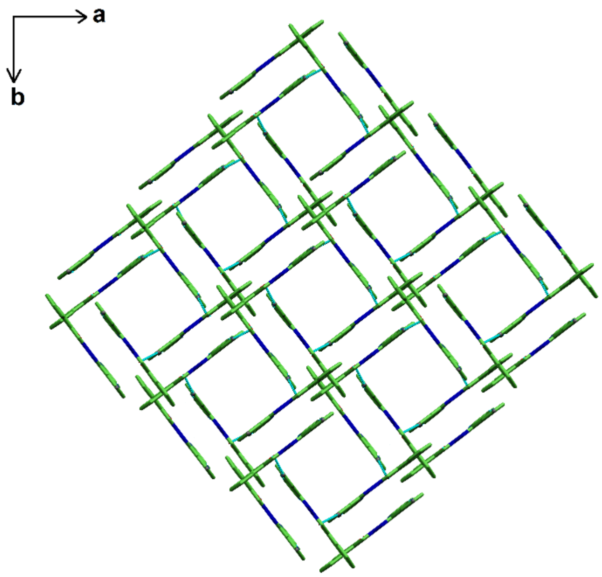

Figure S19. The supramolecular 2D packing diagram of the QNH showing the propagation via H–bonding and short ring–interactions, forms a 2D sheet like structure.

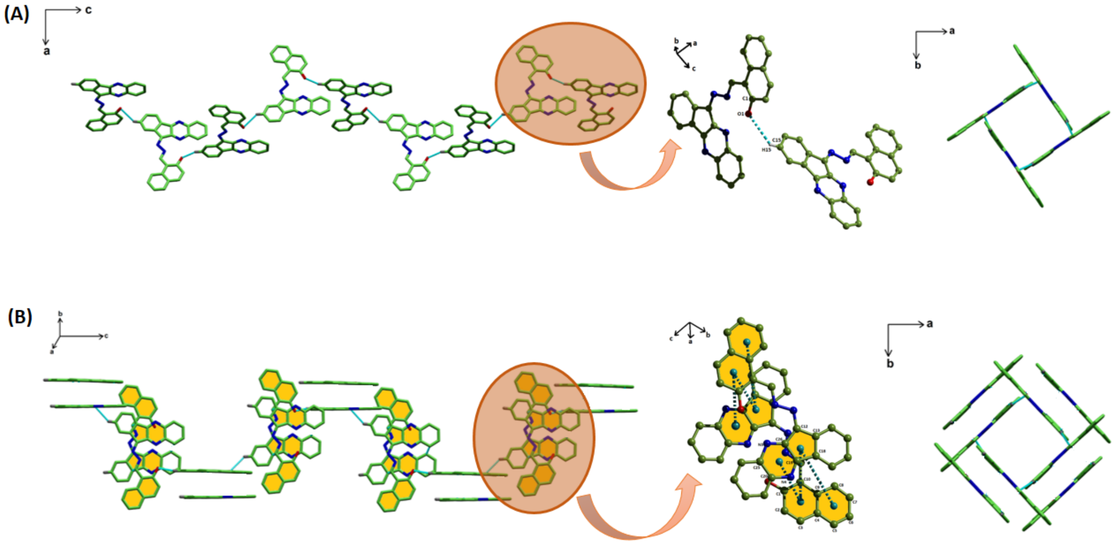

Figure S20. The supramolecular 1D packing diagram of QNH via (A)H–bonding interaction along b axis with enlarged version and c axis view of the interaction ; (B) short ring–interactions with enlarged version and c axis view of the interaction.

Table S1. Colorimetric Cu^{2+} sensing receptors found in the literature.

Ligand structure	Solvent	Detection limit (Absorption)	Ref.
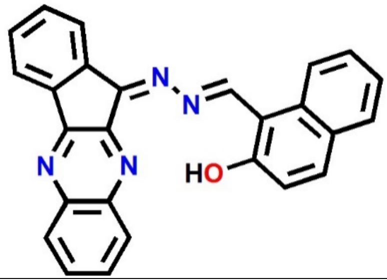	HEPES buffer : CH_3CN (3:7;v/v)	0.45 µM	This work
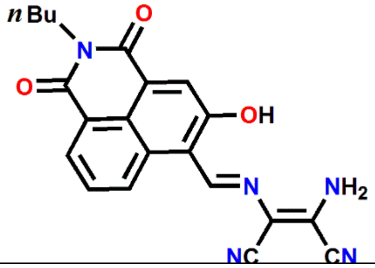	DMSO	1.6 µM	[1]
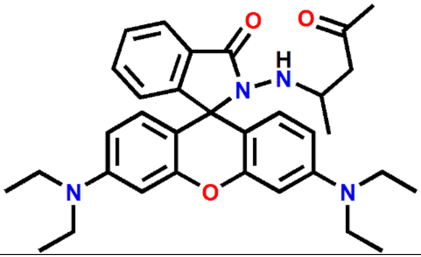	EtOH/ HEPES buffer (9:1; v/v)	2 µM	[2]
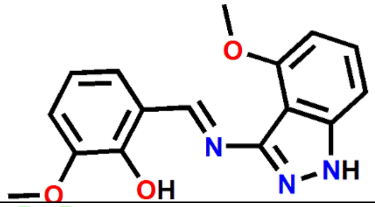	Bis-tris buffer/DMSO (7:3; v/v)	0.14 µM	[3]
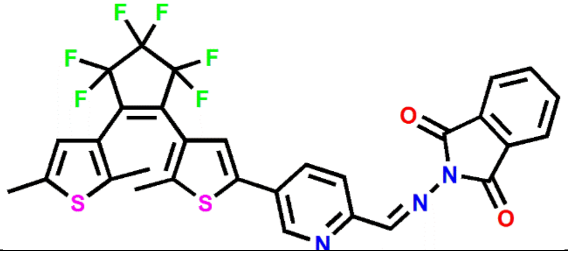	MeOH	0.041 µM	[4]

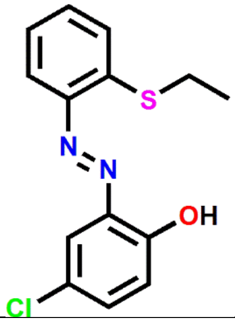	CH_3CN	0.062 µM	[5]
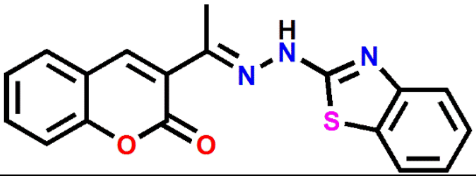	THF: water(9: 1; v/v)	1.54 µM	[6]
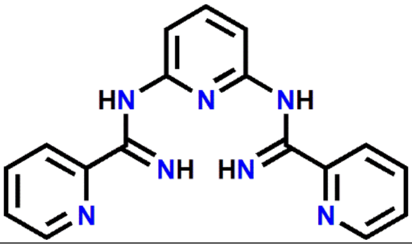	EtOH:H_2O (1:9;v/v)	3.49 µM	[7]
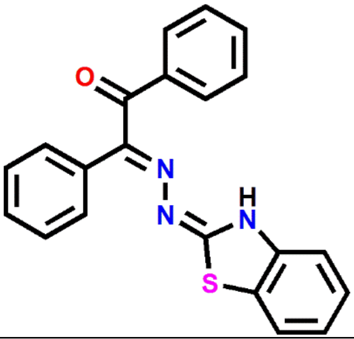	THF–HEPES buffer (9:1;v/v)	10 µM	[8]
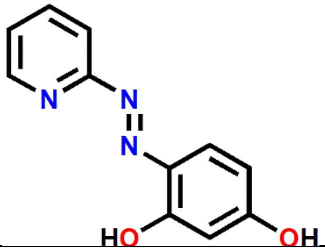	H_2O	0.031 µM	[9]

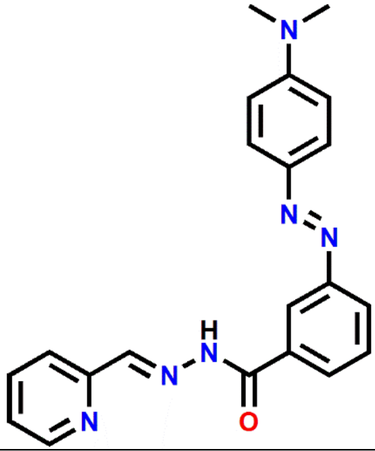	CH_3CN:HEPES buffer (1:1;v/v)	3.68 µM	[10]
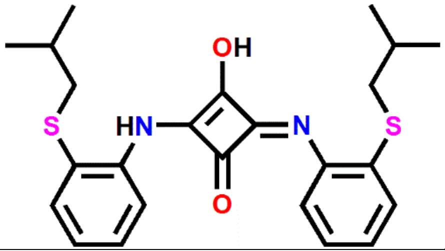	CH_3CN/H_2O (4:1;v/v)	8.06 µM	[11]

Table S2. The crystallographic data for QNH.

CCDC No	2156635
Chem. Formula	$2(C_{26}\,H_{16}\,N_4\,O)$, $C\,H\,Cl_3$
Formula weight	920.22
Crystal color, habit	Orange, rectangular
Temp (K)	296
λ^a/ Å	0.71073
Crystal system	tetragonal
Space group	I 41/a
Unit cell dimensions	
a (Å)	16.455(2)
b(Å)	16.455(2)
c(Å)	33.088(9)
α(deg)	90

β (deg)	90
γ (deg)	90
Volume (Å^3), Z	8959(3),8
Density (mg m^{-3})	1.365
Absorption Coeff(mm^{-1})	0.258
F(000)	3792
Crystal size (mm)	0.19×0.17×0.12
θ range (deg)	1.3–23.7
Limiting indices	–18≤h≤17<br>–18≤k≤18<br>–35≤l≤37
Reflections collected	19954
Unique reflections [R_{int}]	3383(0.093)
Completeness to θ	98.5%(23.7)
Data/restraints/parameters	3383/0/319
GOOF on F^2	1.083
R_1^a, wR_2^bvalues [I > 2σ(I)]	0.1034,0.3079
R_1^a,wR_2^b values (all data)	0.1861,0.3480
Maximum, minimum residual peaks (e Å^{-3})	0.584, -0.400

[a]Graphitemonochromator [b]$R_1 = \Sigma(|F_o| - |F_c|)/\Sigma|F_o|$. [c]$wR_2 = \{\Sigma[w(|F_o|^2 - |F_c|^2)^2]/\Sigma[w(|F_o|^2)^2]\}^{1/2}$

Table S3. The crystallographic data for QNH + Cu^{2+}.

CCDC No	2156636
Chem. Formula	C_{32} H_{30} $Cl_{0.75}$ Cu N_6 $O_{6.50}$
Formula weight	692.75
Crystal color, habit	black bronze, rectangular cuboid
Temp (K)	296
λ^a/ Å	0.71073
Crystal system	triclinic
Space group	*P -1*

Unit cell dimensions	
a (Å)	14.067(4)
b(Å)	15.587(4)
c(Å)	15.601(4)
α(deg)	106.091(7)
β (deg)	91.734(7)
γ (deg)	100.266(8)
Volume ($Å^3$), Z	3222.4(14),4
Density (mg m^{-3})	1.428
AbsorptionCoeff(mm^{-1})	0.795
F(000)	1431
Crystal size (mm)	0.20×0.17×0.11
θ range (deg)	1.36–24.9
Limiting indices	–13≤h≤16<br>–18≤k≤18<br>–18≤l≤18
Reflections collected	27051
Unique reflections [R_{int}]	11126(0.067)
Completeness to θ	99.7% (24.9)
Data/restraints/parameters	11126/0/838
GOOF on F^2	2.051
R_1^a, wR_2^bvalues [I > 2σ(I)]	0.1842, 0.4651
R_1^a,wR_2^b values (all data)	0.2217,0.5019
Maximum, minimum residual peaks (e $Å^{-3}$)	3.32, -1.03

[a]Graphitemonochromator [b]$R_1 = \Sigma(|F_o| - |F_c|)/\Sigma|F_o|$. [c]$wR_2 = \{\Sigma[w(|F_o|^2 - |F_c|^2)^2]/\Sigma[w(|F_o|^2)^2]\}^{1/2}$

Table S4. Analysis of X–I···J (H bonding) interactions in QNH. (Bond lengths (Å), bond angles (°))

I	J	Distance(I···J)	X	∠X–I···J
O1	H15	2.65	C1	104°

Table S5. Analysis of short ring–interactions with Cg···Cg distances (Å) in QNH.

Cg···Cg	Cg···Cg distance (Å)	Slippage(Å)
**Cg1···Cg3**	3.7606(10)	1.382
**Cg3···Cg1**	3.7606(10)	1.333
**Cg1···Cg4**	3.9321(11)	1.824
**Cg4···Cg1**	3.9321(11)	1.741
**Cg2···Cg4**	3.5062(10)	0.327
**Cg4···Cg2**	3.5062(10)	0.406

Cg1: C12 C13 C18 C19 C26

Cg2: N3 C25 C20 N4 C19 C26

Cg3:C1 C2 C3 C4 C9 C10

Cg4:C4 C5 C6 C7 C8 C9

References

[1] I. J. Chang, M. G. Choi, Y. A. Jeong, S. H. Lee, S.-K. Chang, Tetrahedron Lett. **2017**, 58, 474-477.

[2] Y. Hu, J. Zhang, Y.-Z. Lv, X.-H. Huang, S.-l. Hu, Spectrochim. Acta - A: Mol. Biomol. Spectrosc. **2016**, 157, 164-169.

[3] M. S. Kim, J. M. Jung , J. H. Kang , H. M. Ahn , P.-G. Kim, C. Kim ,Tetrahedron **2017**, 73, 4750-4757.

[4] H. Liu, S. Cui, F. Shi, S. Pu , Dyes Pigm. **2019**, 161, 34-43.

[5] P. Roy, S. Das, K. Aich, S. Gharami, L. Patra, T. K. Mondal , J. Indian Chem. Soc. **2017**, 94, 755-760.

[6] M. Pannipara, A. G. A. Sehemi, M. Assiri, A. Kalama , Opt. Mater. **2018**, 79, 255–258.

[7] P. A. Patil, S. Sehlangia, C. P. Pradeep , Sensors International 2021, 2, 100075.

[8] S.-M. Shi, Q. Li, S.-L. Hu, J. Chem. Res. **2019**, 43, 426–430.

[9] H. Tavallali, G. Deilamy-Rad, M. A. Karimi, E. Rahimy, Anal. Biochem. **2019**, 583, 113376.

[10] Y. Zhang, Y.-T. Wang, X.-X. Kang, M. Ge, H.-Y. Feng, J. Han, D.-H. Wang, D.-Z. Zhao, J. Photochem. Photobiol. A: Chem. **2018**, 356, 652-660.

[11] M. Zhao, Y. Zhang, X. Zheng, Z. Li, S. Xu , Inorg. Chem. Commun. **2020**, 121, 108198.
